# *Aureobasidium melanogenum*: a native of dark biofinishes on oil treated wood

**DOI:** 10.1007/s10482-016-0668-7

**Published:** 2016-02-27

**Authors:** Elke J. van Nieuwenhuijzen, Jos A. M. P. Houbraken, Martin Meijer, Olaf C. G. Adan, Robert A. Samson

**Affiliations:** CBS KNAW Fungal Biodiversity Centre, Uppsalalaan 8, 3584 CT Utrecht, The Netherlands; Section Transport in Permeable Media, Department of Applied Physics, University of Technology Eindhoven, Den Dolech 2, 5600 MB Eindhoven, The Netherlands

**Keywords:** *Aureobasidium pullulans*, Linseed oil, Mould staining, Pine, Sustainable, Wood protection

## Abstract

The genus *Aureobasidium,* which is known as a wood staining mould, has been detected on oil treated woods in the specific stain formation called biofinish. This biofinish is used to develop a new protective, self-healing and decorative biotreatment for wood. In order to understand and control biofinish formation on oil treated wood, the occurrence of different *Aureobasidium* species on various wood surfaces was studied. Phenotypic variability within *Aureobasidium* strains presented limitations of morphological identification of *Aureobasidium* species. PCR amplification and Sanger sequencing of ITS and *RPB2* were used to identify the culturable *Aureobasidium* species composition in mould stained wood surfaces with and without a biofinish. The analysed isolates showed that several *Aureobasidium* species were present and that *Aureobasidium melanogenum* was predominantly detected, regardless of the presence of a biofinish and the type of substrate. *A.melanogenum* was detected on wood samples exposed in the Netherlands, Cameroon, South Africa, Australia and Norway. ITS-specific PCR amplification, cloning and sequencing of DNA extracted from biofinish samples confirmed results of the culturing based method: *A. melanogenum* is predominant within the *Aureobasidium* population of biofinishes on pine sapwood treated with raw linseed oil and the outdoor placement in the Netherlands.

**Electronic supplementary material:**

The online version of this article (doi:10.1007/s10482-016-0668-7) contains supplementary material, which is available to authorized users.

## Introduction

*Aureobasidium* are wood staining fungi, in particular on wood situated outdoors above the ground (Dickinson 1972; Dix and Webster 1995; Bardage 1998; Schmidt 2006; Gobakken and Westin 2008). The interest in *Aureobasidium* has recently increased, because of its role in the formation of biofinishes on wood (Sailer et al. 2010; van Nieuwenhuijzen et al. 2013; van Nieuwenhuijzen et al. 2015; Filippovych et al. 2015). The term biofinish was introduced for a uniform dark mould covering which emerged outdoors on oil treated wood (van Nieuwenhuijzen et al. 2015). Although the protection mechanism and durability of this biofinish is still under investigation, biofinished wood is considered to be an appealing biocide-free construction material that has the advantage of also having self-healing properties.

Until now it is unknown which *Aureobasidium* species participates in the biofinish formation and whether a biofinish is composed of more than a single species. Although *Aureobasidium* has been isolated from many organic and inorganic substrates and geographical locations (Zalar et al. 2008; Slepecky and Starmer 2009; Gaur et al. 2010), the ubiquity of the specific species is unknown. The geographical location, the combination of wood species and the oil treatment may all have an impact on the species composition of the *Aureobasidium* population in biofinishes. Species-specific behaviour, such as phenotype and physiology (Samson et al. 2010; Houbraken 2013), should be included in future research in order to understand and control dark mould growth on oil treated wood. Therefore insight in the species composition of the biofinish is highly relevant.

The ascomycete genus *Aureobasidium* is a member of the family *Aureobasidiaceae* within the class of the *Dothideomycetes* (Thambugala et al. 2014; Wijayawardene et al. 2014). *Kabatiella* is closely related to *Aureobasidium* based on morphology and phylogeny (Zalar et al. 2008; Bills et al. 2012; Crous et al. 2011; Peterson et al. 2013; Thambugala et al. 2014) and some of these *Kabatiella* species may belong to *Aureobasidium* (Peterson et al. 2013; Thambugala et al. 2014). In addition future studies may result in the transfer of the species *Selenophoma mahoniae* and *Columnosphaeria* (*Discosphaerina*) *fagi* into *Aureobasidium* (Yurlova et al. 1999; Peterson et al. 2013; Thambugala et al. 2014). A well-known *Aureobasidium* species is *Aureobasidium pullulans* (Zalar et al. 2008; Gostinčar et al. 2014). The total number of classified *Aureobasidium* species currently varies per database, for example 38 in MycoBank and 13 in GenBank (October 2015).

Before DNA sequencing was applied in fungal taxonomy, the species classification system was mainly based on physiologic and phenotypic characteristics. In the case of *Aureobasidium,* colony pigmentation was used as a species-specific phenotypic characteristic (Zalar et al. 2008; Peterson et al. 2013). Nowadays, also the genealogical concordance phylogenetic species recognition (GCPSR) concept is commonly applied for species delimitation (Taylor et al. 2000). For species delimitation according to GCPSR multigene phylogenies are required. Next to the large subunit and the internal transcribed spacer regions (incl. 5.8S rDNA) (ITS) more variable genes such as translation elongation factor 1α, β-tubulin and RNA polymerase II- second largest subunit (*RPB2*) have been applied or recommended for phylogenetic analysis of *Aureobasidium* species (Zalar et al. 2008; Manitchotpisit et al. 2009; Peterson et al. 2013; Gostinčar et al. 2014). A phylogeny, including all described genera and species within the *Aureobasidiaceae*, is not yet available.

The ITS locus is assigned as the primary barcode for fungal species (Schoch et al. 2012). A large number of ITS barcode sequences of *Aureobasidium* species is available in public databases, which makes this DNA region a suitable marker to identify *Aureobasidium* (Manitchotpisit et al. 2009). To date, no second fungal barcode has been determined for a reliable *Aureobasidium* identification on species level.

The aim of this study was to explore the *Aureobasidium* species composition of biofinishes on wood. Culturable *Aureobasidium* isolates, retrieved from substrates with and without biofinishes, were identified. The wood species, oil treatments and exposure sites were related to the culturable species composition. Also direct extraction of biofinish DNA, followed by ITS amplification, cloning and sequencing were used to determine the species compositions of biofinishes.

## Materials and methods

### Substrates and outdoor exposure

**Fig. 1:**
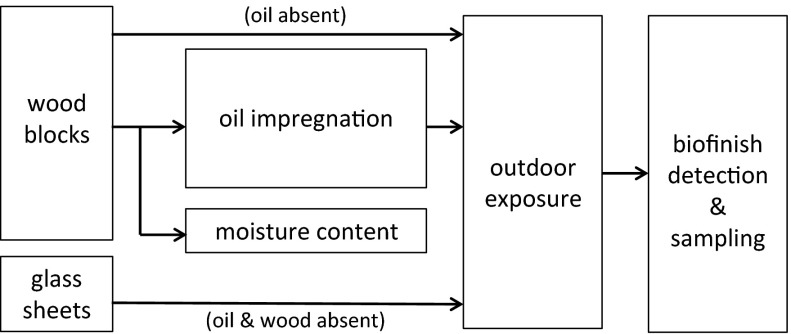
Flowchart illustrating the experimental setup of this study

Nine sample sets were analysed in this study. Each set contained oil treated wood samples. Untreated pine sapwood and glass were also selected for several sample sets (Fig. 1; Table 1), representing oil-free organic and inorganic materials that are associated with *Aureobasidium* growth (Gorbushina and Palinska 1999; Schabereiter-Gurtner et al. 2001; van Nieuwenhuijzen et al. 2015). The amount of different substrates (e.g. wood species, oil type), the geographical location of the outdoor exposure and exposure time was specific for each sample set (Table 1).

**Table 1:** Overview of the sample sets used for outdoor exposure and their characteristics

Samples	Substrate	Number of specimens	Locality	Exposure time
Set 1	Pine sapw. & raw linseed oil	2	Utrecht, The Netherlands	2 weeks
	Glass	2		
Set 2	Pine sapw. & raw linseed oil	2	Utrecht, The Netherlands	5 weeks
	Glass	2		
Set 3	Pine sapw. & raw linseed oil	2	Utrecht, The Netherlands	5 months
	Glass	2		
Set 4	Spruce & raw linseed oil	1	Utrecht, The Netherlands	12 months
	Ilomba & raw linseed oil	1		
	Pine sapw. & raw linseed oil	1		
	Pine sapw. & olive oil	1		
	Pine sapw. & stand oil	1		
	Untreated pine sapw	1		
Set 5	Pine sapw. & raw linseed oil	1	Utrecht, The Netherlands	1.5 years
	Pine sapw. & olive oil	1		
	Pine sapw. & stand linseed oil	1		
	Ilomba & olive oil	1		
	Spruce & olive oil	1		
Set 6	Spruce & raw linseed oil	1	Douala, Cameroon	8 months
	Ilomba & raw linseed oil	1		
	Pine sapw. & raw linseed oil	1		
	Pine sapw. & olive oil	1		
	Untreated pine sapw	1		
Set 7	Same materials as set 6		Johannesburg, South Africa	1.7 years
Set 8	Same materials as set 6		Adelaide, Australia	1.5 years
Set 9	Same materials as set 6		Ås, Norway	2 years

The wood species tested were pine (*Pinus sylvestris*) sapwood, spruce (*Picea abies*) and ilomba (*Pycnanthus angolensis*). No specific sapwood or heartwood selection was made for the latter two species. The surfaces of the wood samples were planed. The samples measured 5 cm (longitudinal axis), × 2.5 cm × 1.5 cm, except the specimens of set 4 which measured 10 cm × 14 cm × 2 cm. Glass sheets (Fisher Scientific) measured 10 cm × 10 cm × 0.3 cm.

Three different vegetable oil types were used to impregnate the wood specimens: raw linseed oil (Vereenigde Oliefabrieken; iodine value 183 and 0.81 % free fatty acids), olive oil (two brands: in case of sample set 4 unfiltered olive oil of 100 % Carolea olive, Calabrië EV Bio 2013; for the other sample sets Carbonel, extra vierge, iodine value 82 and 0.34 % free fatty acids), and stand linseed oil (Vliegenthart, viscosity P45). To determine the average moisture content right before impregnation, additional untreated test pieces of wood were dried at 105 °C. The moisture content of these wood pieces was up to 12 %. The impregnation of the small specimens (5 cm × 2.5 cm × 1.5 cm) was carried out using a vacuum time of 30 min at −1 bar followed by 1 h pressure of 8 bar. A vacuum time of 1 h at −1 bar followed by 2 h pressure of 8 bar was used for the larger specimens of sample set 4.

The wood samples of set 1–3 were steamed twice with hot air for 20 min on two consecutive days (European Standard 1996; Fritsche and Laplace 1999). The glass sheets were cleaned with alcohol and autoclaved before outdoor exposure. No sterilisation method was applied to the wood samples of set 4–9.

Five sites, located in different countries (Table 1) were used for outdoor exposure. The samples from set 4 remained outdoors during the biomass removal. Specifications on outdoor exposure and handling procedures were described in van Nieuwenhuijzen et al. (2015).

### Biofinish assessment

The samples of set 5 and 7–9 have previously been evaluated for biofinish formation in the study of van Nieuwenhuijzen et al. (2015). This method consisted of visual analysis of the stain coverage on the surface as well as in situ spectrophotometer measurements of the pigmentation. In short a biofinish was assigned when the stain coverage was above 90 % and the pigmentation, expressed by triplets as used in the sRGB colour space, met the following criteria: all the red (R), green (G) and blue (B) values were below 82 and the difference between two values of a single RGB triplet was below 20. In comparison the RGB triplet of ultimate black was [0,0,0] and ultimate white was [255,255,255]. The biofinish assessment was also applied on the samples of set 6. The presence of a biofinish on the wood samples of set 1–4 was determined according to the surface coverage part of the biofinish assessment. A full biofinish assessment of the samples of set 4 was performed three months after fungal isolation.

### Collection of *Aureobasidium* isolates

Within a sample set, up to two specimens per substrate were used for isolation (Table 1). The swab sampling method as described in van Nieuwenhuijzen et al. (2015) was used to collect biomass. Biomass suspensions were plated on malt extract agar (MEA) supplemented with penicillin and streptomycin (P/S) and on dichloran 18 % glycerol agar (DG18). The formulation of the agar media were according to Samson et al. (2004) and the plates were incubated at 25 °C for 14 days. A selection of the colonies, that phenotypically resembled *Aureobasidium*, was transferred to new MEA plates. The phenotypic characteristics used to determine *Aureobasidium* colonies: fast growing, yeast like colonies with an irregular edge, either white/pale pink coloured colonies mostly with a black centre and/or sectors or black coloured colonies with a small white boundary; white aerial hyphae sometimes present. Isolates of the selected colonies were deposited in the working collection of the Department of Applied and Industrial Mycology (DTO) housed at the CBS-KNAW Fungal Biodiversity Centre.

### Phenotypic diversity of *Aureobasidium* strain DTO 217-G5

A large phenotypic variation within the *Aureobasidium* colonies on agar plates was observed during isolation. The isolate DTO 217-G5 (= CBS 140241) was used to study the phenotypic variability of a single strain. It was selected as a representative of the black cultures obtained from oil treated wood in the initial stage of biofinish formation. At first biomass was obtained of the edge of a 7 days old colony on MEA and washed in ultrapure water twice before dilution in ultrapure water. This dilution was combined with 10 × Yeast Nitrogen Base (Difco Laboratories 1998) with no additional carbon source and transferred to a shake flask. Due to the limited amount of carbon, the strain was cultivated in a nutrient limited and therefore stressful environment. After 24 h of shaking at 175 rpm at 25 °C, a serial dilution was made of the cell suspension and plated on oatmeal agar. After 5 days of incubation at 25 °C, four phenotypically diverse colony forming units (CFU’s) were selected as parental colonies and inoculated on MEA P/S (first MEA P/S inoculation). After incubation each colony was transferred to a new MEA P/S plate in triplicate. These colonies on the new plates were again transferred to MEA P/S three times in succession. Phenotypically diverse areas were selected for the biomass transfers. Photos were made and ITS sequences generated (as described below) of the colonies of the first and last inoculation on MEA P/S.

### DNA extraction, amplification and sequencing

Isolates were grown on MEA plates prior to DNA extraction. DNA was extracted using the Ultraclean Microbial DNA isolation kit (MoBio Laboratories, Carlsbad, CA, USA) according to the manufacturer’s instructions. The ITS and *RPB2* fragments were amplified using the primer pairs V9G (de Hoog and Gerrits van den Ende 1998) & LS266 (Masclaux et al. 1995) and RPB-PenR1 & RPB-PenR2 (Manitchotpisit et al. 2009). The PCR reactions were performed according to van Nieuwenhuijzen et al. (2015). The *RPB2*-PCR program differed by a primer annealing at 54 °C for 60 s. The amplified DNA fragments were sequenced and assembled as described in Yilmaz et al. (2014). Generated sequences are deposited in GenBank. The accession numbers of the new outdoor isolates are included in Table 2.

**Table 2:** Outdoor isolates obtained in this study and the GenBank accession numbers of their ITS and *RPB2* sequences

Isolate		GenBank accession no.		Isolate		GenBank accession no.	
DTO no.	CBS no.	ITS	*RPB2*	DTO no.	CBS no.	ITS	*RPB2*
DTO 212-D8	–	KT693505	KT693748	DTO 317-A6	–	KT693616	KT693859
DTO 212-F1	–	KT693506	KT693749	DTO 317-A7	–	KT693617	KT693860
DTO 212-G1	–	KT693507	KT693750	DTO 317-A8	–	KT693618	KT693861
DTO 212-I7	–	KT693508	KT693751	DTO 317-A9	–	KT693619	KT693862
DTO 213-A2	–	KT693509	KT693752	DTO 317-B1	–	KT693620	KT693863
DTO 213-A9	–	KT693510	KT693753	DTO 317-B2	–	KT693621	KT693864
DTO 214-C3	–	KT693511	KT693754	DTO 317-B3	–	KT693622	KT693865
DTO 214-C4	–	KT693512	KT693755	DTO 317-B4	–	KT693623	KT693866
DTO 214-C8	–	KT693513	KT693756	DTO 317-B5	–	KT693624	KT693867
DTO 214-D1	–	KT693514	KT693757	DTO 317-B6	–	KT693625	KT693868
DTO 214-D8	–	KT693515	KT693758	DTO 317-B8	–	KT693626	KT693869
DTO 214-D9	–	KT693516	KT693759	DTO 317-B9	–	KT693627	KT693870
DTO 228-C6	–	KT693517	KT693760	DTO 317-C1	–	KT693628	KT693871
DTO 212-H1	–	KT693518	KT693761	DTO 317-C2	–	KT693629	KT693872
DTO 212-H2	–	KT693519	KT693762	DTO 277-B5	–	KT693630	KT693873
DTO 214-E2	–	KT693520	KT693763	DTO 277-B6	–	KT693631	KT693874
DTO 214-E3	–	KT693521	KT693764	DTO 277-B7	–	KT693632	KT693875
DTO 214-F3	–	KT693522	KT693765	DTO 277-B8	–	KT693633	KT693876
DTO 214-F4	–	KT693523	KT693766	DTO 277-B9	–	KT693634	KT693877
DTO 214-G4	–	KT693524	KT693767	DTO 277-C1	–	KT693635	KT693878
DTO 214-G5	–	KT693525	KT693768	DTO 277-C2	–	KT693636	KT693879
DTO 214-I2	–	KT693526	KT693769	DTO 277-C3	–	KT693637	KT693880
DTO 214-I4	–	KT693527	KT693770	DTO 277-C4	–	KT693638	KT693881
DTO 214-I5	–	KT693528	KT693771	DTO 277-C5	–	KT693639	KT693882
DTO 215-B3	–	KT693529	KT693772	DTO 277-C6	–	KT693640	KT693883
DTO 215-B9	–	KT693530	KT693773	DTO 277-C7	–	KT693641	KT693884
DTO 215-C2	–	KT693531	KT693774	DTO 277-G5	–	KT693642	KT693885
DTO 215-C3	–	KT693532	KT693775	DTO 277-G6	–	KT693643	KT693886
DTO 215-D8	–	KT693533	KT693776	DTO 277-F4	140247	KT693644	KT693887
DTO 215-E3	–	KT693534	KT693777	DTO 277-F5	140248	KT693645	KT693888
DTO 217-F3	–	KT693535	KT693778	DTO 277-F6	–	KT693646	KT693889
DTO 217-F4	–	KT693536	KT693779	DTO 277-F7	–	KT693647	KT693890
DTO 217-F5	–	KT693537	KT693780	DTO 277-F8	–	KT693648	KT693891
DTO 217-G4	140240	KT693538	KT693781	DTO 277-F9	–	KT693649	KT693892
DTO 217-G5	140241	KT693539	KT693782	DTO 277-G1	–	KT693650	KT693893
DTO 217-H2	–	KT693540	KT693783	DTO 277-G2	–	KT693651	KT693894
DTO 218-A8	–	KT693541	KT693784	DTO 277-G3	–	KT693652	KT693895
DTO 218-B9	–	KT693542	KT693785	DTO 277-G4	–	KT693653	KT693896
DTO 218-D1	–	KT693543	KT693786	DTO 227-C6	–	KT693654	KT693897
DTO 218-F5	–	KT693544	KT693787	DTO 227-C7	–	KT693655	KT693898
DTO 218-F7	–	KT693545	KT693788	DTO 227-C8	–	KT693656	KT693899
DTO 218-G1	–	KT693546	KT693789	DTO 227-D3	–	KT693657	KT693900
DTO 218-G4	–	KT693547	KT693790	DTO 227-D4	–	KT693658	KT693901
DTO 218-G6	–	KT693548	KT693791	DTO 227-D5	–	KT693659	KT693902
DTO 218-G8	–	KT693549	KT693792	DTO 227-D7	140249	KT693660	KT693903
DTO 218-H6	–	KT693550	KT693793	DTO 227-D8	–	KT693661	KT693904
DTO 218-H8	–	KT693551	KT693794	DTO 227-E3	–	KT693662	KT693905
DTO 218-I1	–	KT693552	KT693795	DTO 227-E4	–	KT693663	KT693906
DTO 228-D1	–	KT693553	KT693796	DTO 227-E6	–	KT693664	KT693907
DTO 218-I3	140242	KT693554	KT693797	DTO 227-E7	–	KT693665	KT693908
DTO 218-I4	140243	KT693555	KT693798	DTO 227-E8	–	KT693666	KT693909
DTO 219-A4	–	KT693556	KT693799	DTO 285-D3	–	KT693667	KT693910
DTO 219-B9	–	KT693557	KT693800	DTO 285-D4	–	KT693668	KT693911
DTO 219-D5	–	KT693558	KT693801	DTO 285-D5	–	KT693669	KT693912
DTO 219-E8	–	KT693559	KT693802	DTO 296-E8	140250	KT693670	KT693913
DTO 219-E9	–	KT693560	KT693803	DTO 285-D6	–	KT693671	KT693914
DTO 219-G2	–	KT693561	KT693804	DTO 285-D7	140251	KT693672	KT693915
DTO 219-H1	–	KT693562	KT693805	DTO 285-D8	–	KT693673	KT693916
DTO 219-I3	–	KT693563	KT693806	DTO 285-D9	–	KT693674	KT693917
DTO 219-B8	–	KT693564	KT693807	DTO 285-E1	140252	KT693675	KT693918
DTO 219-F9	–	KT693565	KT693808	DTO 285-E2	140253	KT693676	KT693919
DTO 219-B2	–	KT693566	KT693809	DTO 285-E3	–	KT693677	KT693920
DTO 232-D6	–	KT693567	KT693810	DTO 285-E4	140254	KT693678	KT693921
DTO 232-D7	–	KT693568	KT693811	DTO 296-F6	–	KT693679	KT693922
DTO 232-E6	–	KT693569	KT693812	DTO 296-G3	–	KT693680	KT693923
DTO 232-E7	–	KT693570	KT693813	DTO 296-G4	–	KT693681	KT693924
DTO 232-H8	–	KT693571	KT693814	DTO 296-G5	140255	KT693682	KT693925
DTO 232-I1	–	KT693572	KT693815	DTO 296-G6	140256	KT693683	KT693926
DTO 232-I8	–	KT693573	KT693816	DTO 285-E5	140257	KT693684	KT693927
DTO 232-I9	–	KT693574	KT693817	DTO 285-E6	140258	KT693685	KT693928
DTO 233-A1	–	KT693575	KT693818	DTO 285-E7	–	KT693686	KT693929
DTO 233-A7	–	KT693576	KT693819	DTO 285-E8	–	KT693687	KT693930
DTO 233-A8	–	KT693577	KT693820	DTO 296-F7	–	KT693688	KT693931
DTO 233-C1	–	KT693578	KT693821	DTO 296-F8	–	KT693689	KT693932
DTO 233-C6	–	KT693579	KT693822	DTO 296-F9	140259	KT693690	KT693933
DTO 233-E3	–	KT693580	KT693823	DTO 296-G1	140260	KT693691	KT693934
DTO 233-F6	–	KT693581	KT693824	DTO 301-G5	–	KT693692	KT693935
DTO 233-F8	–	KT693582	KT693825	DTO 301-G6	–	KT693693	KT693936
DTO 233-G4	–	KT693583	KT693826	DTO 301-G9	140261	KT693694	KT693937
DTO 233-H9	–	KT693584	KT693827	DTO 301-H1	–	KT693695	KT693938
DTO 233-I5	–	KT693585	KT693828	DTO 301-H2	–	KT693696	KT693939
DTO 234-A2	–	KT693586	KT693829	DTO 301-H3	–	KT693697	KT693940
DTO 234-B7	–	KT693587	KT693830	DTO 300-I2	140262	KT693698	KT693941
DTO 234-C7	–	KT693588	KT693831	DTO 300-I3	140263	KT693699	KT693942
DTO 234-D7	–	KT693589	KT693832	DTO 300-I4	–	KT693700	KT693943
DTO 234-E6	–	KT693590	KT693833	DTO 300-I5	–	KT693701	KT693944
DTO 234-F4	–	KT693591	KT693834	DTO 301-F7	140264	KT693702	KT693945
DTO 234-G2	–	KT693592	KT693835	DTO 301-F8	–	KT693703	KT693946
DTO 234-G9	–	KT693593	KT693836	DTO 301-F9	140265	KT693704	KT693947
DTO 316-G9	–	KT693594	KT693837	DTO 300-I8	–	KT693705	KT693948
DTO 316-H1	–	KT693595	KT693838	DTO 300-I9	–	KT693706	KT693949
DTO 316-H2	–	KT693596	KT693839	DTO 301-A6	–	KT693707	KT693950
DTO 316-H3	–	KT693597	KT693840	DTO 301-A7	–	KT693708	KT693951
DTO 316-H4	–	KT693598	KT693841	DTO 301-A8	–	KT693709	KT693952
DTO 316-H5	–	KT693599	KT693842	DTO 301-F4	140266	KT693710	KT693953
DTO 316-H6	140244	KT693600	KT693843	DTO 302-E1	140267	KT693711	KT693954
DTO 316-H7	140245	KT693601	KT693844	DTO 302-E2	–	KT693712	KT693955
DTO 316-H8	–	KT693602	KT693845	DTO 302-E3	–	KT693713	KT693956
DTO 316-I1	140246	KT693603	KT693846	DTO 302-E9	–	KT693714	KT693957
DTO 316-I2	–	KT693604	KT693847	DTO 302-F1	140268	KT693715	KT693958
DTO 316-I3	–	KT693605	KT693848	DTO 302-F2	140269	KT693716	KT693959
DTO 316-I4	–	KT693606	KT693849	DTO 302-F7	–	KT693717	KT693960
DTO 316-I5	–	KT693607	KT693850	DTO 302-F8	–	KT693718	KT693961
DTO 316-I6	–	KT693608	KT693851	DTO 302-H8	–	KT693719	KT693962
DTO 316-I7	–	KT693609	KT693852	DTO 302-H9	–	KT693720	KT693963
DTO 316-I9	–	KT693610	KT693853	DTO 302-I1	–	KT693721	KT693964
DTO 317-A1	–	KT693611	KT693854	DTO 302-I2	–	KT693722	KT693965
DTO 317-A2	–	KT693612	KT693855	DTO 302-G3	–	KT693723	KT693966
DTO 317-A3	–	KT693613	KT693856	DTO 302-H1	–	KT693724	KT693967
DTO 317-A4	–	KT693614	KT693857	DTO 302-H2	–	KT693725	KT693968
DTO 317-A5	–	KT693615	KT693858	DTO 302-H3	–	KT693726	KT693969

### Phylogenetic analysis and identification of isolates

Reference strains of species which were used to generate a benchmark for the molecular identification of the *Aureobasidium* isolates are listed in Table 3. The GenBank accession numbers of the sequences are included in the table, except for the sequences of the *Aureobasidium thailandense* strains generated by Peterson et al. (2013; TreeBASE SN4236). The ITS and *RPB2* sequence data sets were aligned using the program Muscle within MEGA version 5 (Tamura et al. 2011). Maximum Likelihood (ML) analysis was performed using MEGA. The number of bootstrap replicates was set on 1000. *Sydowia polyspora* CBS 750.71 was selected as outgroup. The isolates were identified based on the clustering in the phylogenetic trees with the type and other representative strains. A bootstrap value of 70 % or more was considered as moderated support for the identification of clades.

**Table 3:** *Aureobasidium* and related fungal strains used for molecular identification

Species name	Strain no.	Source	Locality	GenBank assesion no.	
				ITS	*RPB2*
*Aureobasidium leucospermi*	CBS 130593 (epiT)	Leaves of *Leucospermum conocarpodendron*	South Africa	KT693727	KT693970
*Aureobasidium melanogenum*	CBS 105.22 (T)	–	–	KT693729	KT693972
*Aureobasidium melanogenum*	CBS 110374	Public fountain	Thailand, Bangkok	KT693728	KT693971
*Aureobasidium namibiae*	CBS 147.97 (T)	Dolomitic marble	Namibia, Namib Desert	KT693730	KT693973
*Aureobasidium proteae*	CBS 114273 (epiT)	Leaves of *Protea* *cv. ‘Sylvia’*	South Africa	KT693731	KT693974
*Aureobasidium proteae*	CBS 111973	Leaves of *Protea* *cv. ‘Sylvia’*	South Africa	KT693732	KT693975
*Aureobasidium pullulans*	CBS 584.75 (NT)	*Vitis vinifera*, fruit	France, Beaujolais, Beaujeu	KT693733	KT693976
*Aureobasidium pullulans*	CBS 100280	Salt pan	Slovenia	KT693734	KT693977
*Aureobasidium subglaciale*	CBS 123387 (T)	Subglacial ice from sea water	Norway, Svalbard, Kongsvegen	KT693735	KT693978
*Aureobasidium subglaciale*	CBS 123388	Glacial ice from sea water	Norway, Svalbard, Kongsvegen	KT693736	KT693979
*Aureobasidium thailandense*	CBS 133856, NRRL 58539 (T)	Leaves of *Cerbera odollum*	Thailand, Nakhonratchasima	GenBank no. absent; TreeBASE SN4236	
*Aureobasidium thailandense*	CBS 133857, NRRL 58543	Wood surface	Thailand, Prachuapkhirikhan	GenBank no. absent; TreeBASE SN4236	
*Columnosphaeria fagi (Discosphaerina fagi)*	CBS 171.93	Leaf of *Populus*	United Kingdom	KT693737	KT693980
*Kabatiella bupleuri*	CBS 131304 (isoT)	Dead flower stems, *Bupleurum gibraltarium*	Spain, Granada, Embalse de Canales	KT693738	KT693981
*Kabatiella bupleuri*	CBS 131303	Dead flower stems, *Bupleurum gibraltarium*	Spain, Granada, Presa de Quentar	KT693739	KT693982
*Kabatiella caulivora*	CBS 242.64	*Trifolium incarnatum*	U.S.A., Oregon	KT693740	KT693983
*Kabatiella harpospora*	CBS 122914	Stems and leaves of *Viscum album*	Spain, Madrid, Robledo de Chavela	KT693741	absent
*Kabatiella lini*	CBS 125.21 (T)	*Linum usitatissimum*	United Kingdom	KT693742	KT693984
*Kabatiella microsticta*	CBS 114.64	*Hemerocallis* sp.	The Netherlands, Wageningen	KT693744	KT693986
*Kabatiella microsticta*	CBS 342.66	*Convallaria majalis*, dying leave	Germany	KT693743	KT693985
*Kabatiella zeae*	CBS 767.71	Leaf of *Zea mays*	Germany, Kiel-Kitzeberg	KT693745	absent
*Selenophoma mahoniae*	CBS 388.92	Leaf of *Mahonia repens*	U.S.A., Colorado	KT693746	KT693987
*Sydowia polyspora*	CBS 750.71	*Pinus strobus*, twig	Canada, Quebec; Lac Normand	KT693747	KT693988

*T* ex-type strain, *NT* ex-neotype strain, *epiT* ex-epitype strain, *isoT* ex-isotype strain

### PCR, cloning and sequencing of biofinish DNA

ITS-specific cloning libraries were made of biofinishes of two types of substrates of set 5 in triplicate: pine sapwood & raw linseed oil (library PRL.1–PRL.3) and pine sapwood & olive oil (library PO.1–PO.3). An area of 2.5 cm × 2.5 cm of the upper surface of a specimen was scratched with a scalpel and DNA was extracted of the obtained biomass. The DNA extraction method, ITS primers and PCR-program were identical to the method described above. The PCR master mixes with ITS primers were prepared with the GoTaq Long PCR Master Mix (Progema) according to the manufacturer’s instructions. The PCR products were purified with the QIAquick PCR purification kit. Following the manufacturer’s instructions, 45 ng of PCR products was ligated and cloned (pGEM^®^-T Easy Vector Systems) into an *Escherichia coli* plasmid library. After growing ITS containing competent cells on plate, colonies were aseptically transferred to 10 μl demineralised water. PCR reactions were performed in 25 μl reaction mixtures containing 3 μL aliquots with ITS DNA, 2.5 μl PCR buffer, 2 μl MgCl2 (25 mM), 11 μl demineralised sterile water, 5 μL dNTP (1 mM), 0.50 μl of each primer (10 µM) and 0.5 μl Taq polymerase (5 U/μL, Bioline). The ITS-PCR program, sequencing, and assembling were similar to the previously described method. Assembled ITS sequences were generated of 62–69 cloned colonies per wood sample library. The ITS sequences of the cloning libraries were screened against the non-redundant NCBI database, using the program BLASTN. Sequences resulting in hits with an identity of 97 % or more compared to *Aureobasidium* sequences of the database were used for phylogenetic analysis. Sequences were submitted to GenBank (Table 4).

**Table 4:** All sequences from the cloning library identified as *Aureobasidium* and their corresponding GenBank accession numbers

ITS clone	Accession no.	ITS clone	Accession no.	ITS clone	Accession no.	ITS clone	Accession no.
PRL.1.05	KT693388	PRL.2.02	KT693421	PRL.2.87	KT693456	PRL.3.80	KT693487
PRL.1.06	KT693389	PRL.2.03	KT693422	PRL.3.02	KT693457	PRL.3.82	KT693488
PRL.1.09	KU671015	PRL.2.05	KT693423	PRL.3.06	KT693458	PRL.3.83	KT693489
PRL.1.21	KT693390	PRL.2.07	KT693424	PRL.3.08	KT693459	PO.1.13	KT693490
PRL.1.25	KT693391	PRL.2.08	KT693425	PRL.3.09	KT693460	PO.1.37	KT693491
PRL.1.26	KT693392	PRL.2.22	KT693426	PRL.3.16	KU671021^a^	PO.1.45	KT693492
PRL.1.27	KT693393	PRL.2.23	KT693427	PRL.3.17	KT693461	PO.1.50	KT693493
PRL.1.30	KT693394	PRL.2.25	KT693428	PRL.3.19	KU671022^a^	PO.1.59	KT693494
PRL.1.31	KT693395	PRL.2.26	KT693429	PRL.3.21	KT693462	PO.1.68	KT693495
PRL.1.32	KU671016	PRL.2.27	KT693430	PRL.3.22	KT693463	PO.1.73	KT693496
PRL.1.33	KT693396	PRL.2.29	KT693431	PRL.3.23	KT693464	PO.1.75	KU671024^a^
PRL.1.34	KT693397	PRL.2.34	KT693432	PRL.3.24	KT693465	PO.1.81	KT693497
PRL.1.38	KU671017	PRL.2.35	KT693433	PRL.3.25	KT693466	PO.2.78	KT693498
PRL.1.39	KT693398	PRL.2.36	KT693434	PRL.3.28	KT693467	PO.3.05	KT693499
PRL.1.50	KT693399	PRL.2.37	KT693435	PRL.3.31	KT693468	PO.3.24	KT693500
PRL.1.51	KT693400	PRL.2.38	KT693436	PRL.3.32	KT693469	PO.3.68	KT693501
PRL.1.53	KT693401	PRL.2.39	KT693437	PRL.3.36	KT693470	PO.3.69	KT693502
PRL.1.54	KT693402	PRL.2.43	KT693438	PRL.3.37	KT693471	PO.3.81	KT693503
PRL.1.58	KT693403	PRL.2.44	KT693439	PRL.3.39	KT693472	PO.3.88	KT693504
PRL.1.61	KT693404	PRL.2.47	KT693440	PRL.3.42	KT693473		
PRL.1.62	KT693405	PRL.2.49	KT693441	PRL.3.43	KT693474		
PRL.1.67	KT693406	PRL.2.50	KT693442	PRL.3.44	KT693475		
PRL.1.69	KT693407	PRL.2.51	KT693443	PRL.3.47	KT693476		
PRL.1.70	KT693408	PRL.2.62	KT693444	PRL.3.51	KT693477		
PRL.1.71	KT693409	PRL.2.63	KT693445	PRL.3.54	KT693478		
PRL.1.74	KT693410	PRL.2.64	KT693446	PRL.3.55	KT693479		
PRL.1.75	KT693411	PRL.2.65	KT693447	PRL.3.56	KT693480		
PRL.1.76	KT693412	PRL.2.70	KT693448	PRL.3.57	KT693481		
PRL.1.77	KT693413	PRL.2.73	KT693449	PRL.3.65	KT693482		
PRL.1.78	KT693414	PRL.2.74	KU671018	PRL.3.66	KT693483		
PRL.1.79	KT693415	PRL.2.76	KT693450	PRL.3.68	KU671019		
PRL.1.81	KT693416	PRL.2.78	KT693451	PRL.3.70	KU671023		
PRL.1.82	KT693417	PRL.2.80	KT693452	PRL.3.71	KU671020^a^		
PRL.1.85	KT693418	PRL.2.82	KT693453	PRL.3.73	KT693484		
PRL.1.86	KT693419	PRL.2.83	KT693454	PRL.3.74	KT693485		
PRL.1.88	KT693420	PRL.2.86	KT693455	PRL.3.78	KT693486		

^a^Sequences were trimmed to remove chimeric parts

## Results

### Biofinish assessment

All wood samples of sample set 3–9 showed dark discolorations, but a biofinish was only established on a few samples (Table 5). Biofinishes were detected on specific samples exposed for more than one year at the sample site in the Netherlands (sample set 4 and 5): pine sapwood samples treated with raw linseed oil or olive oil and spruce and ilomba samples treated with olive oil. Furthermore, biofinishes were detected on the pine sapwood sample treated with olive oil that was exposed in South Africa (sample set 7) and the pine sapwood sample treated with raw linseed oil that was exposed in Norway (sample set 9).

**Table 5:** Overview of the (number of) *Aureobasidium* isolates per substrate (with or without biofinish) of each sample set. (− = not relevant)

Sample set	Substrate	Biofinish present	Number of isolates	DTO isolate code (CBS number added when available)
Set 1	Pine sapw. & raw lins. oil	No	13	212-D8, 212-F1, 212-G1, 212-I7, 213-A2, 213-A9, 214-C3, 214-C4, 214-C8, 214-D1, 214-D8, 214-D9, 228-C6
	Glass	–	17	212-H1, 212-H2,214-E2, 214-E3, 214-F3, 214-F4, 214-G4, 214-G5, 214-I2, 214-I4, 214-I5, 215-B3, 215-B9, 215-C2, 215-C3, 215-D8, 215-E3
Set 2	Pine sapw. & raw lins. oil	No	19	217-F3, 217-F4, 217-F5, 217-G4 (CBS 140240), 217-G5 (CBS 140241), 217-H2, 218-A8, 218-B9, 218-D1, 218-F5, 218-F7, 218-G1, 218-G4, 218-G6, 218-G8, 218-H6, 218-H8, 218-I1, 228-D1
	Glass	–	13	218-I3 (CBS 140242), 218-I4 (CBS 140243), 219-A4, 219-B9, 219-D5, 219-E8, 219-E9, 219-G2, 219-H1, 219-I3, 219-B8, 219-F9, 219-B2
Set 3	Pine sapw. & raw lins. oil	No	19	232-D6, 232-D7, 232-E6, 232-E7, 232-H8, 232-I1, 232-I8, 232-I9, 233-A1, 233-A7, 233-A8, 233-C1, 233-C6, 233-E3, 233-F6, 233-F8, 233-G4, 233-H9, 233-I5
	Glass	–	8	234-A2, 234-B7, 234-C7, 234-D7, 234-E6, 234-F4, 234-G2, 234-G9
Set 4	Spruce & raw lins. oil	No	5	316-G9, 316-H1, 316-H2, 316-H3, 316-H4
	Ilomba & raw lins. oil	No	4	316-H5, 316-H6 (CBS 140244), 316-H7 (CBS 140245), 316-H8
	Pine sapw. & raw lins. oil	Yes	7	316-I1 (CBS 140246), 316-I2, 316-I3, 316-I4, 316-I5, 316-I6, 316-I7
	Pine sapw. & olive oil	Yes	8	316-I9, 317-A1, 317-A2, 317-A3, 317-A4, 317-A5, 317-A6, 317-A7
	Pine sapw. & stand oil	No	5	317-A8, 317-A9, 317-B1, 317-B2, 317-B3
	Untreated pine sapw	No	7	317-B4, 317-B5, 317-B6, 317-B8, 317-B9, 317-C1, 317-C2
Set 5	Pine sapw. & stand lins. oil	No	7	277-B5, 277-B6, 277-B7, 277-B8, 277-B9, 277-C1, 277-C2
	Ilomba & olive oil	Yes	7	277-C3, 277-C4, 277-C5, 277-C6, 277-C7, 277-G5, 277-G6
	Spruce & olive oil	Yes	10	277-F4 (CBS 140247), 277-F5 (CBS 140248), 277-F6, 277-F7, 277-F8, 277-F9, 277-G1, 277-G2, 277-G3, 277-G4
Set 6	Spruce & raw lins. oil	No	3	227-C6, 227-C7, 227-C8
	Ilomba & raw lins. oil	No	3	227-D3, 227-D4, 227-D5
	Pine sapw. & raw lins. oil	No	2	227-D7 (CBS 140249), 227-D8
	Pine sapw. & olive oil	No	2	227-E3, 227-E4
	Untreated pine sapw	No	3	227-E6, 227-E7, 227-E8
Set 7	Spruce & raw lins. oil	No	4	285-D3, 285-D4, 285-D5, 296-E8 (CBS 140250)
	Ilomba & raw lins. oil	No	4	285-D6, 285-D7 (CBS 140251), 285-D8, 285-D9
	Pine sapw. & raw lins. oil	No	9	285-E1 (CBS 140252), 285-E2 (CBS 140253), 285-E3, 285-E4 (CBS 140254), 296-F6, 296-G3, 296-G4, 296-G5 (CBS 140255), 296-G6 (CBS 140256)
	Pine sapw. & olive oil	Yes	4	285-E5 (CBS 140257), 285-E6 (CBS 140258), 285-E7, 285-E8
	Untreated pine sapw	No	4	296-F7, 296-F8, 296-F9 (CBS 140259), 296-G1 (CBS 140260)
Set 8	Spruce & raw lins. oil	No	2	301-G5, 301-G6
	Ilomba & raw lins. oil	No	4	301-G9 (CBS 140261), 301-H1, 301-H2, 301-H3
	Pine sapw. & raw lins. oil	No	7	300-I2 (CBS 140262), 300-I3 (CBS 140263), 300-I4, 300-I5, 301-F7 (CBS 140264), 301-F8, 301-F9 (CBS 140265)
	Pine sapw. & olive oil	No	2	300-I8, 300-I9
	Untreated pine sapw	No	4	301-A6, 301-A7, 301-A8, 301-F4 (CBS 140266)
Set 9	Spruce & raw lins. oil	No	3	302-E1 (CBS 140267), 302-E2, 302-E3
	Ilomba & raw lins. oil	No	3	302-E9, 302-F1 (CBS 140268), 302-F2 (CBS 140269)
	Pine sapw. & raw lins. oil	Yes	6	302-F7, 302-F8, 302-H8, 302-H9, 302-I1, 302-I2
	Pine sapw. & olive oil	No	1	302-G3
	Untreated pine sapw	No	3	302-H1, 302-H2, 302-H3

### Collection of *Aureobasidium* isolates

The number of isolates used in this study varied per substrate of each set (Table 5). These isolates were obtained from CFU’s on agar plates after culturing biomass of the substrates. They represent a small number of all the CFU’s which phenotypically resembled *Aureobasidium* species. For example 7–10 isolates were studied per sample in set 5 (Table 5), while the total amount of the counted *Aureobasidium* CFU’s on MEA and DG18 was up to 9 × 10^3^ per sampled surface (van Nieuwenhuijzen et al. publication in progress).

### Phenotypic diversity of *Aureobasidium* strain DTO 217-G5

**Fig. 2:**
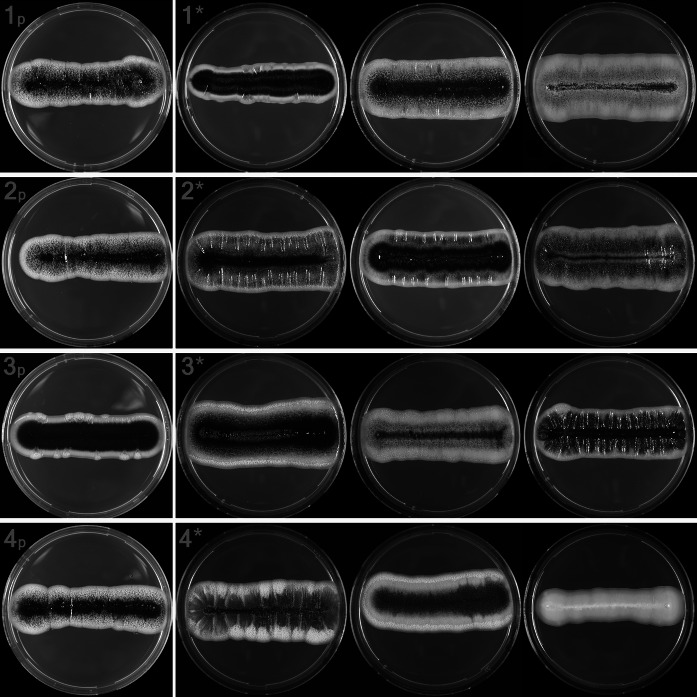
Macromorphology of various inoculations of *A. melanogenum* strain DTO 217-G5; 1p–4p: first inoculation on MEA P/S of four single CFU’s, grown at 25 °C for 6 days; 1*–4*: inoculation of the same four single CFU’s on MEA P/S after four consecutive transfers, grown at 25 °C for 7 days in triplicate

The macromorphology of various inoculations of DTO 217-G5 (= CBS 140241) were compared to study the limitations of a phenotypic classification method for *Aureobasidium* species. The ITS barcode of isolate DTO 217-G5 and all its inoculations were identical. Based on this data, the isolate was identified as *Aureobasidium melanogenum* (Supplementary Data Fig. 1). The studied colonies of DTO 217-G5 (Fig. 2) were considered to be pure single strains since they were obtained as CFU’s after plating a serial diluted yeast-like cell suspension. After the first transfer of four of the CFU’s, which had different phenotypic characteristics on OA, to MEA P/S plates all colonies showed dark pigmentation and aerial hyphae in the margin and a more or less equal colony diameter (at 6 days of incubation). The colony texture, degree of pigmentation and mycelial production varied. The colonies after another three consecutive times of transfer and incubation showed more variation in their macromorphology. Although almost all examined cultures showed dark pigmentation, the degree varied widely and was even absent in one culture. Also the colony surface area and appearance varied. Some colonies produced aerial hyphae at the margin and the degree of hyphal production varied between isolates. Furthermore, the slimy appearance of the colonies which is described as cultural characteristic of *A. melanogenum* (Zalar et al. 2008), was also absent in some cultures.

### Identification of *Aureobasidium* isolates

**Fig. 3:**
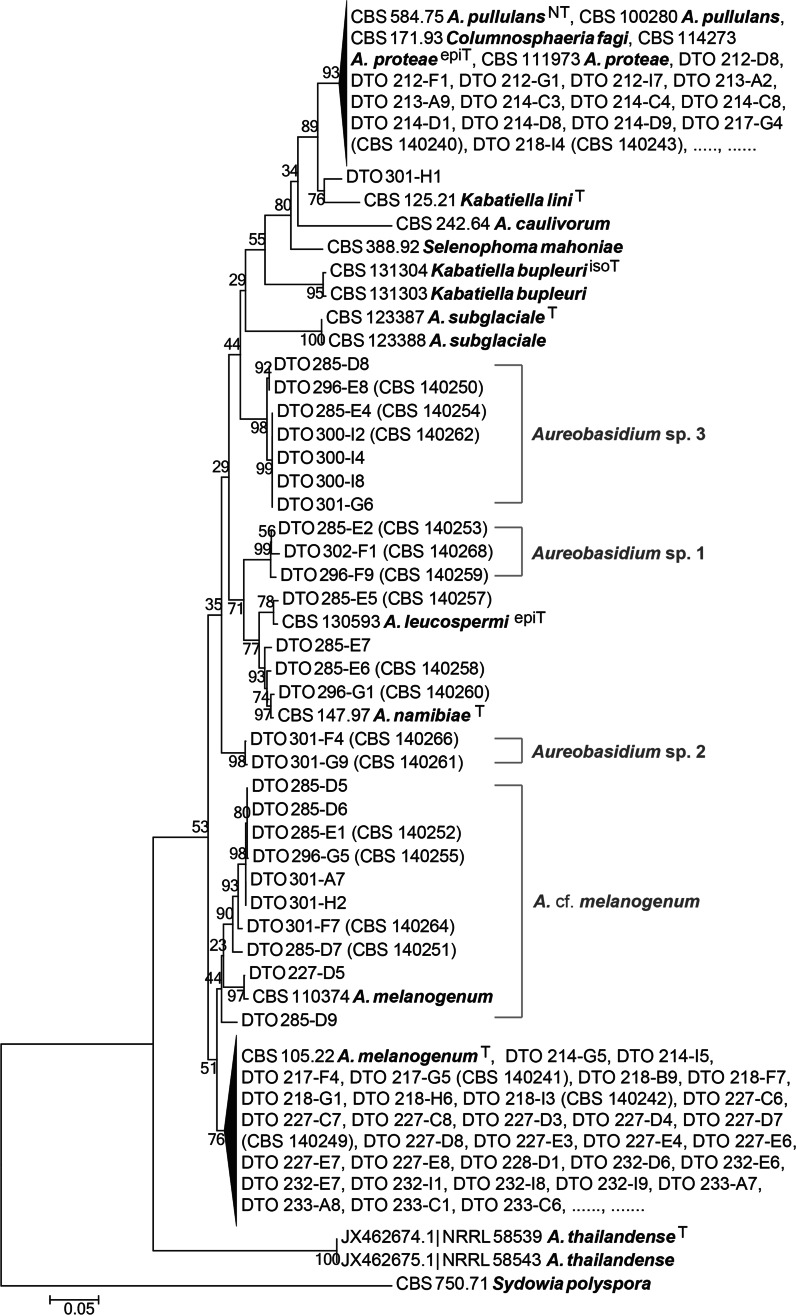
Maximum Likelihood tree of concatenated ITS and *RPB2* sequences from outdoor *Aureobasidium* isolates and the classified reference strains. The *bar* indicates the number of substitutions per site. *T* ex-type strain, *NT* ex-neotype strain, *epiT* ex-epitype strain, *isoT* ex-isotype strain

The majority of all 222 sequenced *Aureobasidium* isolates could be unambiguously identified (Fig. 3). *Aureobasidium proteae* and *Columnosphaeria fagi* resided in a clade with *A. pullulans* (Fig. 3) and are considered as synonyms of *A. pullulans*. The majority of the strains clustered together with the type of *A. melanogenum* (CBS 105.22^T^). Eleven strains had similar sequences as the type of *A. melanogenum*; however, these strains couldn’t be confidentially resolved in the *A. melanogenum* clade (bootstrap values below 7 0 %, Fig. 3). The sequence variation could be fully attributed to the *RPB2* part of the concatenated sequences. These strains were therefore identified as *Aureobasidium* confer (cf.) *melanogenum.* Three clades with moderate bootstrap support (Fig. 3) did not contain any type or other reference strains and the isolates in these groups were tentatively named *Aureobasidium* sp. 1, sp. 2 and sp. 3. Sequences of strains named *K. microsticta,K. harpospora*, and *K. zeae* were excluded from the *Aureobasidium* phylogenetic overview. *Kabatiella microsticta* was represented by two strains that were placed in two far apart clades in the phylogenetic tree while none of these strains were classified as type strain. The latter two *Kabatiella* species were closer related to the outgroup than to the other *Aureobasidium* species.

Interestingly, 18 of the 222 *Aureobasidium* isolates had ambiguous nucleotide sites in their *RPB2* sequences. Eleven of these isolates were identified as *A. melanogenum*, one as *A. pullulans* and six as *Aureobasidium* sp. 1. The bootstrap values were above 70 % (Supplementary Data Fig. 2).

### Aureobasidium species composition on stained wood surfaces

**Fig. 4:**
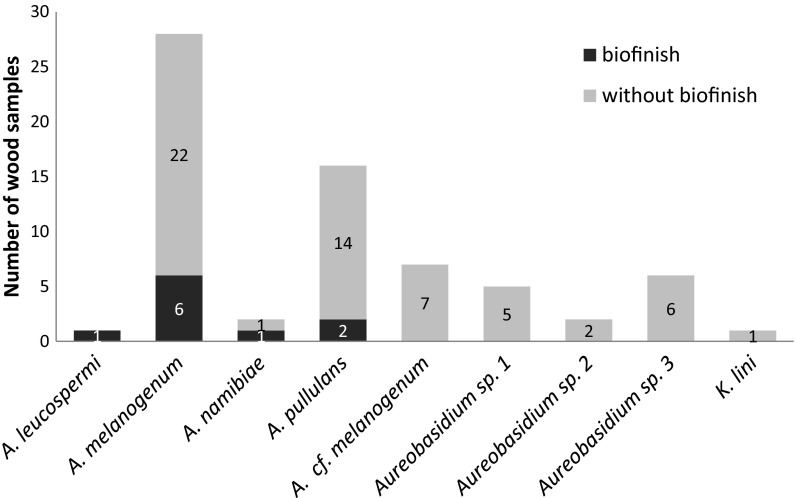
*Aureobasidium* species detected on the long-term outdoor exposed wood samples of sample sets 3–9. The total number of wood samples with a biofinish was six and without biofinish 27

Isolates from the biofinish containing wood samples revealed that all six biofinishes contained *A.melanogenum* (Fig. 4). Other detected species were *A. leucospermi*, *A. namibiae* and *A. pullulans*. The isolates consisted of 42 *Aureobasidium* colonies that were selected after culturing biomass from biofinish containing wood. 81 % of these isolates were identified as *A. melanogenum*.

Isolates from the 27 wood specimens, which contained visual mould staining but did not have a biofinish, showed that 80 % of these wood specimens contained *A.melanogenum.* In addition to this species, also *A.* cf. *melanogenum, A. namibiae*, *A. pullulans, K. lini*, and *Aureobasidium* sp. 1, sp. 2, and sp. 3 were detected on the mould stained wood samples without biofinish (Fig. 4). These isolates consisted of 110 *Aureobasidium* colonies that were selected after culturing biomass from the sample surfaces. 60 % of these isolates were identified as *A. melanogenum*.

#### Impact of different wood and (oil-) treatments on the species composition

*A. melanogenum* was detected on 21 of the 25 wood samples of sets 4 and 6–9 after exposure at the five different sites. *A. melanogenum* was (one of) the most detected species for each substrate. Per substrate 3–6 other species were found. The species were identified as *A. leucospermi*, *A. namibiae*, *A. pullulans*, *A.* cf. *melanogenum*, *Aureobasidium* sp. 1, *Aureobasidium* sp. 2, *Aureobasidium* sp. 3 or *K. lini*. These species were in most cases detected on one to two samples per substrate.

#### Impact of exposure sites on the species occurrence

**Fig. 5:**
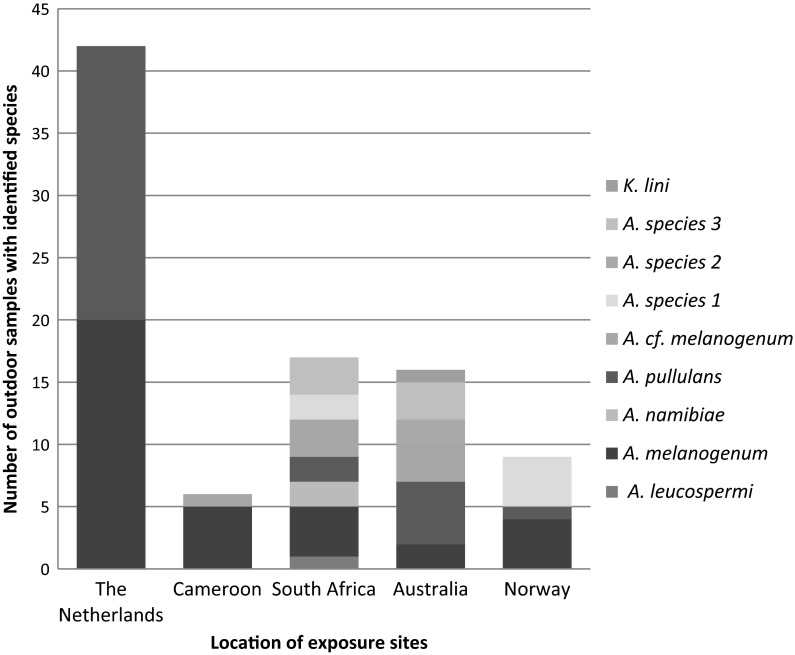
Identified *Aureobasidium* species obtained from samples of sample sets 1–9 sorted by exposition site

*A. melanogenum* was detected in the sample sets exposed outdoors in the Netherlands, Cameroon, South Africa, Australia and Norway (Fig. 5). Other *Aureobasidium* species were detected as well, but were not obtained from all locations (Fig. 5). This outcome could be influenced by the limited number of isolates analysed per location. For example, 13–25 isolates were obtained from samples exposed at sites outside the Netherlands (Table 5).

Samples exposed in Australia and South Africa contained the highest *Aureobasidium* species diversity (Fig. 5). In the Netherlands, only *A. melanogenum* and *A. pullulans* isolates were detected, despite the relatively high number of substrate types (9) and identified isolates (149). This indicates that the detectable species diversity of outdoor placed substrates is influenced by the location of the exposure site.

#### Aureobasidium species composition on oil treated wood in time

**Fig. 6:**
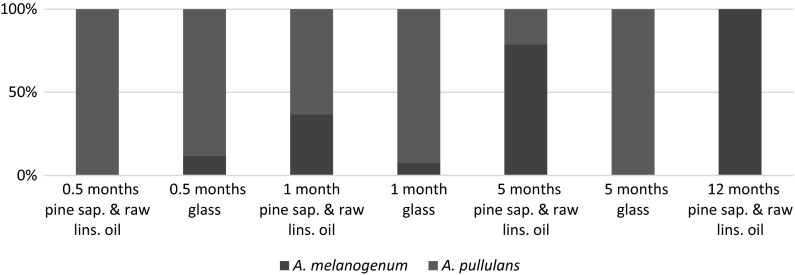
Composition of *Aureobasidium* isolates obtained from outdoor exposed oil treated pine sapwood samples or glass sheets

The isolates from the pine sapwood samples treated with raw linseed oil (sample sets 1–4) showed that the number of colonies identified as *A. pullulans* decreased over time and the number of colonies identified as *A. melanogenum* increased over time (Fig. 6). At 5 and 12 months of outdoor exposure of the samples, when mould staining on the wood surface was present, the majority of the corresponding analysed colonies were identified as *A. melanogenum*. This in contrast to the results of the analysed colonies isolated from the reference material glass and the pine sapwood samples that had a shorter exposure time. More than 80 % of a colony set retrieved from glass was identified as *A. pullulans* regardless the exposition time.

### PCR, *cloning* and sequencing of biofinish DNA

In order to analyse the *Aureobasidium* species composition of biofinishes on wood without a cultivation step, cloning libraries were generated of the DNA of six biofinishes. Each of the six cloning libraries contained clones with ITS DNA that belonged to several genera. In all libraries at least one sequence was identified as *Aureobasidium* by BLASTN on the NCBI database. Most of the sequences obtained from clones with *Aureobasidium* DNA clustered together in the phylogenetic trees with either the two *A. melanogenum* or the *A. pullulans* reference strains (bootstrap values above 63 %). A few *Aureobasidium* sequences (PRL.1.31, PRL.1.69, PRL3.16, PRL.3.19, PRL.3.70 and PO1.75) could not be identified on species level (Table 6), because they did not cluster with any of the known species. Further investigation revealed that these sequences contained parts of more than 100 nucleotides that differed largely from the reference strains.

**Table 6:** *Aureobasidium* sequences of ITS-specific clones generated from biofinishes of set 5, identified to species level

Biofinish	Substrate	Number of clones identified as *Aureobasidium*				Predominant species
		Total	*A. melanogenum*	*A. pullulans*	Unidentified	
PRL.1	Pine sapwood & raw linseed oil	35	33		2	*A. melanogenum*
PRL.2		37	37			
PRL.3		38	35		3	
PO.1	Pine sapwood & olive oil	9	7	1	1	Unclear
PO.2		1	1			
PO.3		6	3	3		

The cloning libraries of the biofinishes on the samples treated with raw linseed oil had more than 50 % of all 62–69 clones per library identified as *Aureobasidium*. The predominant species of these cloning libraries was *A. melanogenum* (Table 6). The predominant species within the *Aureobasidium* DNA of biofinishes obtained from pine sapwood samples treated with olive oil remains unclear. Firstly the amount of clones identified as *Aureobasidium* per library was much lower, varying from 1 to 8 clones, and secondly one library showed the number of clones identified as *A. pullulans* to be equal to the ones identified as *A. melanogenum* (Table 6).

## Discussion

### Identification of *Aureobasidium* species

#### Morphology

Different phenotypic characteristics are described per *Aureobasidium* species (Zalar et al. 2008; Samson et al. 2010). The deviation of the general macromorphological characteristics of a strain within an *Aureobasidium* species can be explained by degeneration (Zalar et al. 2008) or phenotypic plasticity (Slepecky and Starmer 2009). Morphological changes of fungal strains on culture media after serial transfers have also been observed for other fungal species, for example *Aspergillus flavus* (Horn and Dorner 2002) and *Metarhizium anisopliae* (Ryan et al. 2002). The results in this study of the culturing of strain DTO 217-G5 showed, that phenotypic characteristics of various isolates of a single strain inoculated on the same media can differ widely. Although the original isolate DTO 217-G5 could accidently be a mixture of strains, the studied subcultures of DTO 217-G5 were likely to be pure single strains and they still showed phenotypic diversity. Regardless the phenotypic variation of these cultures, the ITS sequences were identical. This made the use of molecular techniques, instead of morphological characteristics, essential to identify the *Aureobasidium* isolates on species level.

#### Multi-locus DNA analysis

Several isolates had ambiguous nucleotide positions in their *RPB2* sequence. The accidental presence of more than one strain in an examined isolate could explain these ambiguous nucleotide positions. In order to exclude the presence of multiple strains in a single isolate, biomass of an isolate was cultured in liquid media and after plating, separate colony forming units were used for PCR and sequencing; however, ambiguous nucleotide positions remained present in the sequences (unpublished data). Since *RPB2* is regarded as a single-copy protein coding gene (Schmitt et al. 2009; Schoch et al. 2012), more tests are needed to study this phenomenon.

The reference data set for identification of *Aureobasidium* strains contained sixteen different described taxa (Table 3) and thirteen were shown in the *Aureobasidium* phylogenetic overview (Fig. 3). The distant relationship of *K. harpospora* (CBS 122914) to the *Aureobasidium* species and the placement of the *K. microsticta* strains (CBS 114.64 and CBS 342.66) in different phylogenetic clades is in concordance with Zalar et al. (2008) and Bills et al. (2012). The placement of *Kabatiella zeae* CBS 767.71 apart from the other *Aureobasidium* species can be supported by the outcome of a homology search of the ITS sequence of CBS 767.71 on GenBank: the best hit was *Lecythophora* sp. (KF624793.1).

### Composition of *Aureobasidium* species

The composition of *Aureobasidium* species in a biofinish on wood can be studied with different techniques. However, no single technique is available that ensures the exact result (Nevalainen et al. 2015). It is well known that techniques that are based on culturing fungi have limitations that will influence the outcome of the fungal composition (Pitkäranta et al. 2008). For example: (a) conidia might produce more colonies on an agar plate than the same amount of biomass represented by hyphae (Pitt and Hocking 2009), (b) some fungi only grow on specific culturing conditions (Pitt and Hocking 2009; Samson et al. 2010), (c) some fungi are not culturable (Pitkäranta et al. 2011; Dei-Cas et al. 2006; Blackwell 2011) and (d) some species are overgrown by other fungi in mixed samples (Samson et al. 2010). Culture independent techniques, such as targeted cloning and sequencing of DNA regions or next generation amplicon sequencing, have been used increasingly over the last years to study the composition of fungal communities, for example in the area of soil ecology (Orgiazzi et al. 2013; Clemmensen et al. 2013), wood decay (Lindner et al. 2011; van der Wal et al. 2014) and human health (Ghannoum et al. 2010; Findley et al. 2013). Although culture independent techniques are the state of the art, they also have drawbacks. Each step of a DNA based method can introduce a bias. Examples are the differences in efficiency of DNA extraction per fungal species or morphologic structure (Saad et al. 2004; Fredricks et al. 2005) and the differences in efficiency of PCR amplification per species and primer set (Vainio and Hantula 2000; Bellemain et al. 2010). In the case of amplification of the ITS region, the species dependent number of copies of the targeted rDNA region per cell (Vilgalys and Gonzalez 1990; Simon and Weiss 2008; Lindner and Banik 2011) contributes to the different PCR amplification efficiencies. Another example of a bias is the absence of registration of taxa which are relatively scarcely present in a DNA mixture (Adams et al. 2013a; Prakitchaiwattana et al. 2004).

The origin of the fungal isolates, retrieved from the outdoor exposed specimens, is considered to be the concerned exposure site. The natural occurrence of *Aureobasidium* on outdoor exposed materials is well known and the distribution of *Aureobasidium* occurs by wind disturbance (Taylor et al. 2006), water drops (Hudson 1992; Madelin 1995) or insects (Zacchi and Vaughan-martini 2003; Pagnocca et al. 2008). Not only wood has been reported as outdoor substrate (Dix and Webster 1995; Schmidt 2006), but also other organic materials, such as leaves (Andrews et al. 2002; Woody et al. 2007), grapes (Prakitchaiwattana et al. 2004), as well as painted surfaces (Shirakawa et al. 2002; Kelley et al. 2006), plastic (Reynolds 1950; Webb et al. 2000), glass (Gorbushina and Palinska 1999; Schabereiter-Gurtner et al. 2001) and stone (Urzì et al. 2001; Ruibal et al. 2005). It should be noted that several wood samples in this study were subjected to unsterile handling and packaging.

Although analysis of bigger data sets and the use of other methods might generate a more complete view on the species compositions, the results obtained in this study indicated the predominance of *A. melanogenum* within the *Aureobasidium* population of biofinishes using a culture-based method. No deviation was observed among the different substrates of the long term exposed wood samples. The predominance of *A. melanogenum* in biofinishes on pine sapwood treated with raw linseed has been confirmed using a DNA-based method without a cultivation step. In order to confirm the indication that *A. melanogenum* is predominant within the *Aureobasidium* population of biofinishes generated on other substrates or at other exposition sites, more studies are needed. Also the potential predominance of this species within the entire fungal population of biofinishes should be investigated further. Next to *Aureobasidium* other wood stain fungi, such as species of *Cladosporium* and *Sydowia*, are to be expected (Schmidt 2006; Viitanen and Ritschkoff 2011). The use of a next generation sequencing approach, next to the culturing and PCR cloning method as used in this study, will allow a detailed compositional analysis (van Nieuwenhuijzen et al., publication in progress).

### The assets of *A. melanogenum* in biofinish formation

The finding of *A. melanogenum* as native of biofinishes on oil treated wood is a first step in understanding and controlling biofinish formation. More research is recommended on the growth mechanisms of biofinishes. The deposition (natural inoculation), attachment, survival and reproduction of fungal fragments on the oil treated wood surface are all development steps of biofinish growth outdoors.

The natural inoculation of the substrate will be influenced by the natural occurrence of certain species at a specific location. Although *Aureobasidium* has been detected in outdoor air with short-term air sampling (Larsen and Gravesen 1991; Beaumont et al. 1985; Spicer and Gangloff 2005; Pyrri and Kapsanaki-Gotsi 2007) and outdoor located sedimentation plates (Urzì et al. 2001; Adams et al. 2013b), the occurrence of some *Aureobasidium* species seem to depend on the specific outdoor location. Both *A. melanogenum* and *A. pullulans* are widely spread and might be globally present species. *A. melanogenum* isolates in this study originated from five widespread locations and this species has been isolated outdoors by others in South Africa (CBS 131917, isolated by Van der Walt), Japan, Thailand and Norway (Zalar et al. 2008). Strains of *A. pullulans* originated from the Netherlands, South Africa, Australia and Norway (this study) and at least five other countries (Zalar et al. 2008). The absence of this species in the isolates originating from Cameroon could be explained by the relative low number of isolates (Fig. 5). In contrast, the widespread occurrence of various other *Aureobasidium* species is less likely, because the relative high number of isolates from the Netherlands only consisted of *A. melanogenum* and *A. pullulans*.

The results in this study on short term exposed samples indicated that in the first weeks of outdoor exposure *A. pullulans* was more present than *A. melanogenum* on raw linseed oil treated wood as well as on glass. This did not disturb the predominance of *A. melanogenum* in a later stage of the biofinish formation. Especially since composition of *Aureobasidium* species in time seemed different on glass, the dominant influence of developments steps other than deposition seems likely.

Thus far no data has been found as to why *A. melanogenum* is predominant within the *Aureobasidium* population of biofinishes on oil treated wood. It is currently unknown whether *A. melanogenum* is better in attachment, survival and/or reproduction on outdoor wood surfaces than other *Aureobasidium* species.

With respect to attachment: the biosynthesis of pullulan, an extracellular polymeric substance (EPS) adhesive, is described for at least four *Aureobasidium* species (Gostinčar et al. 2014) and also the production of other EPS, such as β-glucan and acidic polysaccharides, by different *Aureobasidium* species is known (Leal-Serrano et al. 1980; Hamada and Tsujisaka 1983; Yurlova and de Hoog 1997; Lotrakul et al. 2013).

Obviously, the production of melanin by *Aureobasidium* seems to be involved in its survival (Rättö et al. 2001; Hernández 2012; Nosanchuk and Casadevall 2003; Ruan et al. 2004; Paolo et al. 2006; Kogej et al. 2007). However, it is currently unknown whether *A. melanogenum* has an overall higher melanin content in comparison to the other species as may be suggested by its name. Genetic evidence, based on the presence of the number of genes possibly related to melanin synthesis, in an *A. melanogenum* strain (CBS 110374) and other full genome-sequenced *Aureobasidium* strains (Gostinčar et al. 2014) does not indicate obvious differences between the *Aureobasidium* species. Not only the amount, but also the type of melanin, that is produced by each species and the impact of different melanin types on specific stressors (e.g. UV, oxidizing agents) needs to be unravelled to understand the role of melanin. This requires an extensive investigation since many complicating factors are involved such as the difference in pigmentation of various isolates of a single *A. melanogenum* strain (Fig. 2), the existence of many other colours besides black in pigments resulting from melanin (Langfelder et al. 2003; Pal et al. 2014), the impact of exposure conditions on the amount of (unspecified) melanin produced by a single strain (Hernández and Evans 2015a, b) and the inability of easy melanin quantification methods, such as spectrophotometric measurements, to determine the type of melanin (Pal et al. 2014).

Next to survival, organisms need to multiply in order to support dark mould staining. Substrates are considered to play a role in this. One of the factors influenced by substrates is the availability of nutrients for fungal growth (van Nieuwenhuijzen et al. 2015). For example, Horvath et al. (1976) presumed that nutrients for *A. pullulans* formation on wood substrates are derived from the wood. Schoeman and Dickinson (1997) also concluded that this species uses nutrients derived from wood, in particularly the products of lignocellulosic photo degradation at weathered wood surfaces. However one should keep in mind that these referred studies were performed before the recognition of *A. pullulans* and *A. melanogenum* as separate species. Next to the wood also additional materials such as oil in the case of biofinishes on oil treated wood (van Nieuwenhuijzen et al. 2013, 2015) or the attracted organic matter such as pollen (Hudson 1992) might be used for growth of *Aureobasidium*. Possibly the nutrients on oil treated wood are more favourable for *A. melanogum* than other *Aureobasidium* species. More research is needed to understand the impact of substrates on the biofinish population.

## Conclusions

The culture based study showed the common presence of *A. melanogenum* in biofinishes that were naturally formed outdoors on oil treated wood. This fungus was also commonly found on wood samples with non-biofinish mould staining. On most of the outdoor exposed wood samples that contained stained surfaces, *A. melanogenum* was isolated, regardless the type of (oil) treatment or wood species. *A.melanogenum* was detected on samples of all five widespread exposure sites. Other *Aureobasidium* species were detected on the wood samples as well, including several potentially new species in the case of the non-biofinish samples. The results indicated that the diversity of culturable *Aureobasidium* species depends on the geographical location of the exposure site. Larger data sets for these and other locations will be required to allow more defined conclusions. ITS-specific PCR, cloning and sequencing of biofinish DNA confirmed the predominance of *A. melanogenum* within the *Aureobasidium* population of biofinishes generated in the Netherlands on pine sapwood samples treated with raw linseed oil. To allow a detailed composition analysis of the entire fungal population of biofinishes, the use of data obtained with culturing, PCR cloning and a next generation sequencing approach is suggested for future works.

## Electronic supplementary material

Below is the link to the electronic supplementary material.

Supplementary material 1 (TIFF 30136 kb)

Supplementary material 2 (TIFF 23305 kb)

Supplementary material 3 (DOCX 13 kb)
