# Supplementary figures and images for: Aureobasidium melanogenum: a native of dark biofinishes on oil treated wood
Source: Antonie Van Leeuwenhoek. 2016 Feb 27;109:661–83. doi: 10.1007/s10482-016-0668-7 (PMC4819947; doi:10.1007/s10482-016-0668-7)

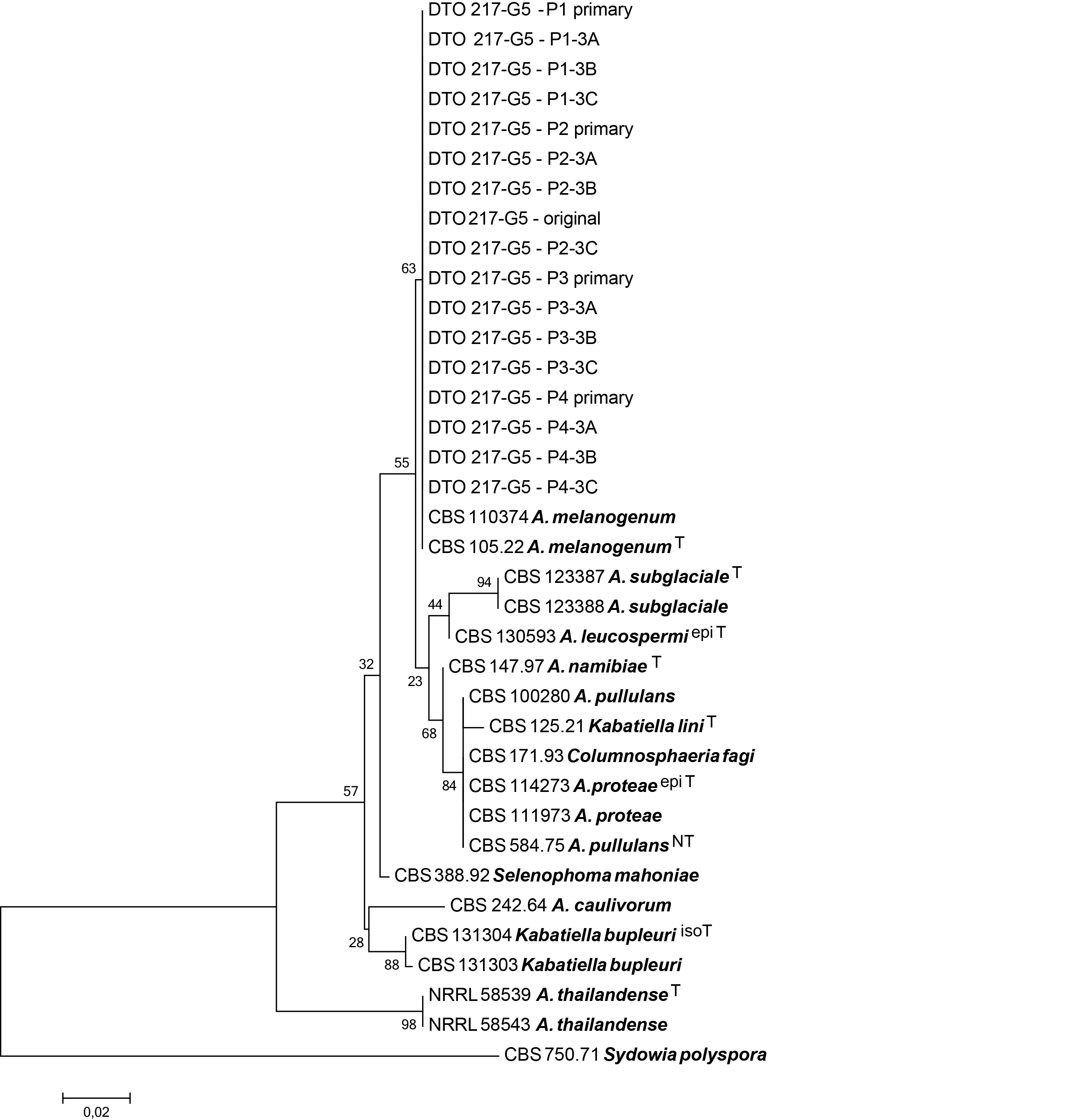

Supplement: Supplementary file 1 — Supplementary material 1 (TIFF 30136 kb) [file 10482_2016_668_MOESM1_ESM.tif]

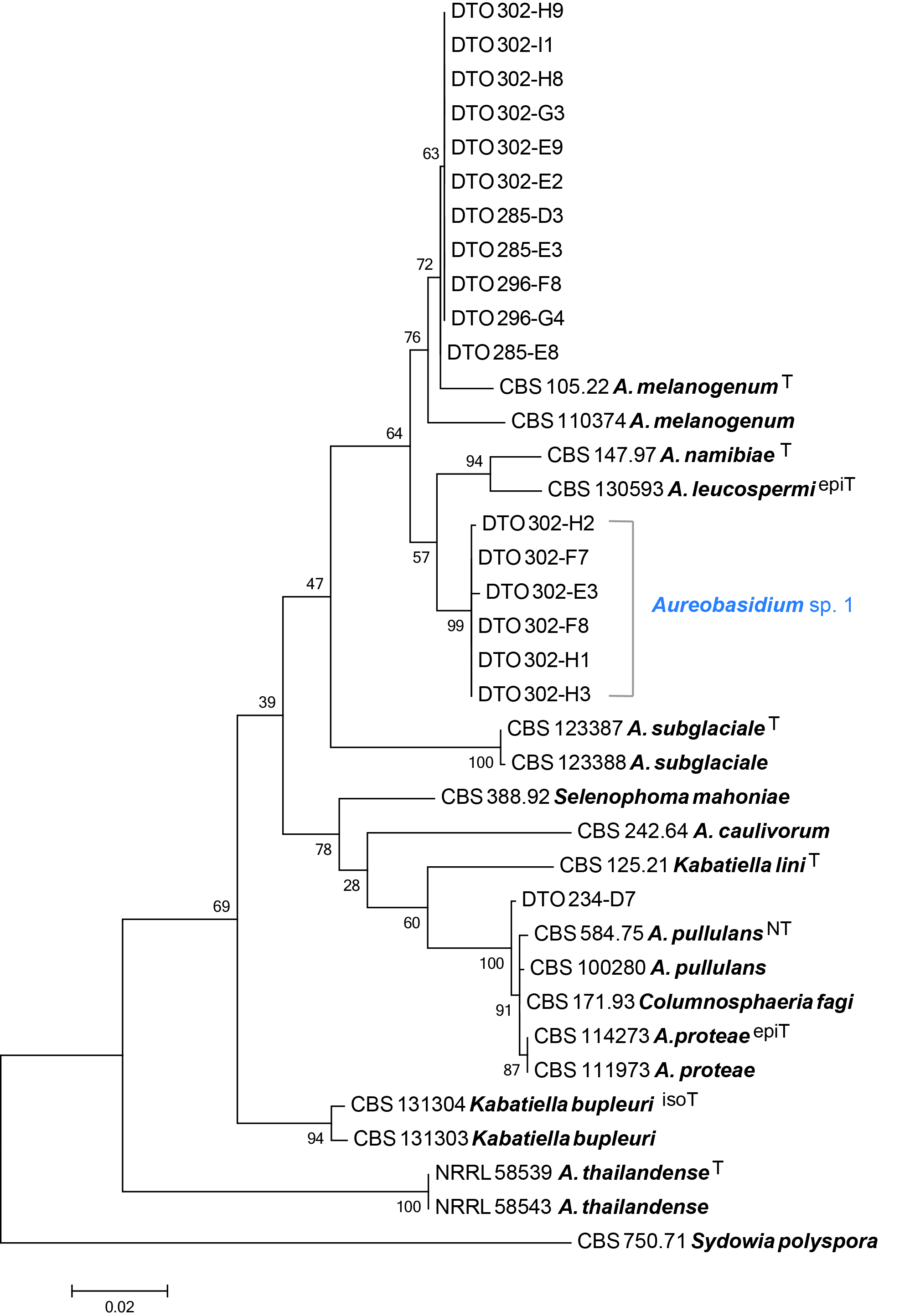

Supplement: Supplementary file 2 — Supplementary material 2 (TIFF 23305 kb) [file 10482_2016_668_MOESM2_ESM.tif]
