# Supplementary material for: Aureobasidium melanogenum: a native of dark biofinishes on oil treated wood
Source: Antonie Van Leeuwenhoek. 2016 Feb 27;109:661–83. doi: 10.1007/s10482-016-0668-7 (PMC4819947; doi:10.1007/s10482-016-0668-7)
Supplement: Supplementary file 3 — Supplementary material 3 (DOCX 13 kb) [file 10482_2016_668_MOESM3_ESM.docx]

# Figure Legends Supplementary data

**Fig. 1** Maximum Likelihood tree based on ITS fragments of strain DTO 217-G5 (= CBS 140241), its subcultures and *Aureobasidium* and related fungal reference strains.

Footnote:

Abbreviations: T = ex-type strain, NT = ex-neotype strain, epiT = ex-epitype strain, isoT= ex-isotype strain

**Fig. 2** Maximum Likelihood tree based on concatenated ITS & *RPB2* sequences from outdoor *Aureobasidium* isolates with ambiguous nucleotide sites and reference strains .

Footnote:

Abbreviations: T = ex-type strain, NT = ex-neotype strain, epiT = ex-epitype strain, isoT= ex-isotype strain
